# Integrating Control-Mastery Theory and EMDR in the treatment of attachment-related trauma: pathogenic beliefs, testing processes, and target selection

**DOI:** 10.3389/fpsyg.2026.1918050

**Published:** 2026-10-12

**Authors:** Ludovica Bedeschi, Margherita Onofri

**Affiliations:** 1 Centro Clinico De Sanctis, Rome, Italy; 2 Pts Psicoterapia Training School, Rome, Italy; 3 University of Arkansas Rome Center, Rome, Italy

**Keywords:** attachment, complex developmental trauma, control mastery theory, Eye Movement Desensitization and Reprocessing (EMDR), pathogenic beliefs, target selection, relational psychotherapy

## Abstract

Attachment-related trauma often develops through repeated relational experiences involving emotional misattunement, role reversal, inconsistent caregiving, and chronic threats to relational security. Such experiences may contribute to pathogenic beliefs concerning dependency, responsibility, emotional expression, and interpersonal safety, while making the identification of clinically meaningful targets for trauma processing particularly complex. This paper proposes a clinical-theoretical integration of Control-Mastery Theory (CMT) and Eye Movement Desensitization and Reprocessing (EMDR) for the treatment of attachment-related trauma. Drawing on the CMT concepts of pathogenic beliefs and testing, we suggest that relational processes within therapy may provide clinically useful information about the beliefs currently organizing the patient’s difficulties. Observation of passive-into-active, transference, and direct tests may help clinicians formulate and refine hypotheses about the relational meanings linking present difficulties to developmental experiences. When integrated with standard AIP-informed history-taking and EMDR target assessment, this information may contribute to the prioritization of candidate attachment-related memories for processing. The paper also introduces the concept of relational readiness as a complementary dimension of EMDR case conceptualization, referring to the extent to which pathogenic relational expectations have become sufficiently observable within the therapeutic relationship to inform target formulation, without replacing established considerations of stabilization, affect tolerance, or readiness for trauma processing. A clinical illustration is presented to demonstrate the proposed sequence from relational testing and disconfirmation to EMDR target formulation and processing. The model is intended as a hypothesis-generating framework rather than an empirically validated treatment protocol, and its incremental clinical value requires systematic investigation.

## 1 Introduction

Attachment-related trauma remains a central focus of clinical research and practice within the EMDR community. Eye Movement Desensitization and Reprocessing (EMDR) is well established as an effective treatment for Post-Traumatic Stress Disorder (PTSD) and other trauma-related conditions (Shapiro, 2001, 2018). However, important conceptual and clinical questions remain regarding its application to developmental and attachment-related trauma. Unlike trauma resulting from a discrete event, attachment-related trauma often emerges through repeated relational experiences between a child and significant caregivers. Patterns such as emotional unavailability, role reversal, chronic caregiver distress, inconsistent responsiveness, and implicit threats to relational security may gradually contribute to enduring beliefs about the self, others, and relationships. Consequently, the patient’s difficulties may not be traceable to a single traumatic memory but may instead reflect cumulative relational experiences whose meanings continue to organize present functioning. A major clinical challenge in attachment-related trauma concerns determining which experiences are most relevant as potential targets for EMDR processing. Patients may report numerous distressing childhood experiences, yet these memories are not necessarily equally relevant to their current difficulties. Some may be highly emotionally salient without being central to present functioning, whereas apparently minor relational experiences may remain clinically important because of the meanings they acquired within the attachment relationship.

The Adaptive Information Processing (AIP) model proposes that symptoms persist when inadequately processed memories continue to influence present experience when triggered (Shapiro, 2001). The model provides a well-established framework for understanding how unresolved traumatic memories contribute to current suffering, but offers less specific guidance for determining which among multiple attachment-related memories should be prioritized in complex developmental presentations. This issue is particularly relevant when patients present with diffuse histories of emotional neglect, role reversal, or chronic relational insecurity rather than a single well-defined traumatic event. Control-Mastery Theory (CMT; Weiss and Sampson, 1986; Weiss, 1993) may offer a useful complementary perspective. CMT proposes that individuals enter treatment with an unconscious plan to overcome pathogenic beliefs developed from adverse developmental experiences. Although these beliefs may originally have been adaptive within the childhood environment, they can continue to constrain emotional and interpersonal functioning after the original circumstances have changed. Within psychotherapy, patients are understood to test these beliefs through interactions with the therapist, seeking experiences that may disconfirm pathogenic expectations and support psychological growth.

The present paper proposes that Control-Mastery Theory may contribute to EMDR case conceptualization in attachment-related trauma by providing a clinically observable pathway from pathogenic beliefs to memory targets. More specifically, we argue that testing processes within the therapeutic relationship render pathogenic beliefs progressively visible to both patient and therapist. As these beliefs become increasingly explicit and begin to undergo relational disconfirmation, the clinician may be better able to formulate and refine hypotheses about the attachment-related experiences that continue to sustain their emotional credibility. In this framework, the therapeutic relationship does not merely provide safety for memory processing; it may also provide clinically relevant information that contributes to hypotheses about which attachment-related memories remain relevant to the patient’s current suffering.

Particular attention will be given to attachment configurations characterized by role reversal and inverted caregiving. In such contexts, children frequently develop pathogenic beliefs organized around responsibility for the emotional wellbeing of others, fears of burdening significant attachment figures, and expectations that the expression of personal needs may produce relational harm. These configurations provide a particularly fruitful context in which to examine the relationship between pathogenic beliefs, testing processes, and EMDR target selection.

Several existing contributions have addressed the challenges of applying EMDR to complex, developmental, and attachment-related trauma. Knipe (2014) described interweave strategies for addressing ego-state conflicts and early relational injuries, while Mosquera and colleagues (Mosquera and Knipe, 2015; Mosquera et al., 2014) emphasized stabilization, pacing, and the management of pervasive relational expectations in complex clinical presentations. Within an EMDR–AEDP integration, Gleiser et al. (2008) explored how affective and relational change processes may complement memory reprocessing in trauma presentations characterized by relational disruption.

Importantly, relational and attachment-informed approaches to EMDR case conceptualization and target identification are not themselves new. Parnell’s (2013) Attachment-Focused EMDR explicitly incorporates attachment history, developmental experiences, and the therapeutic relationship into EMDR treatment, including strategies such as bridging and floatback for identifying relevant developmental targets. More recently, attachment-informed approaches have further emphasized the importance of understanding formative relational experiences within EMDR case formulation rather than organizing treatment exclusively around symptom intensity (Brayne, 2024). Leeds (2021) has also proposed an integrative model of case formulation drawing on the Adaptive Information Processing model, Attachment Theory, and the Theory of Structural Dissociation, in which an integrated understanding of the patient informs treatment planning and target sequencing. More recently, Hase and Brisch (2022) have emphasised the therapeutic relationship as an integral component of EMDR Therapy, including its attachment-related dimensions, a perspective subsequently explored empirically in a survey of EMDR therapists (Hase et al., 2025).

The novelty of the present proposal therefore does not lie in asserting that relational or attachment-related information should inform EMDR case conceptualization or target selection. Rather, it lies in applying the specific conceptual architecture of Control-Mastery Theory to this clinical problem. In particular, CMT offers a functional account of how pathogenic beliefs may become observable through identifiable forms of testing within the therapeutic relationship–including passive-into-active, transference, and direct tests–and how their progressive relational disconfirmation may provide an additional source of information for case formulation. The proposed contribution is therefore narrower and more specific: testing processes are conceptualized as clinically observable relational phenomena that may help formulate and refine hypotheses about the pathogenic beliefs currently organizing the patient’s difficulties and, in conjunction with standard AIP-informed history-taking and target assessment, help prioritize candidate attachment-related memories for EMDR processing.

The aim of this paper is therefore not to propose a new form of EMDR protocol, nor to suggest that EMDR should be subordinated to Control-Mastery Theory. Rather, it seeks to explore how the integration of these two perspectives may contribute to a more precise understanding of attachment-related trauma and to a more clinically informed selection of memory targets within EMDR treatment.

## 2 Attachment-related trauma, role reversal, and the development of pathogenic beliefs

Attachment-related trauma differs in important ways from trauma resulting from discrete catastrophic events. While acute traumatic experiences are often associated with identifiable episodes of threat, attachment trauma typically emerges through repeated interactions within caregiving relationships that shape the child’s expectations about safety, emotional regulation, dependency, and interpersonal relatedness over time. The traumatic impact of these experiences derives not only from what occurred, but from the developmental significance of the relationship in which they occurred.

From the perspective of attachment theory, children depend upon caregivers not merely for physical protection but also for emotional regulation and the construction of coherent internal working models of self and others (Bowlby, 1969/1982, 1988). When caregivers are consistently available, emotionally responsive, and capable of helping the child regulate distress, attachment relationships provide the foundation for psychological security and adaptive development. Conversely, when caregiving relationships are characterized by chronic inconsistency, emotional unavailability, fear, role confusion, or excessive dependence upon the child, developmental adaptation may require the child to organize experience around strategies that preserve the attachment bond at the expense of other developmental needs.

These observations converge with accounts of disorganized attachment and its relational sequelae (Lyons-Ruth et al., 1999; Liotti, 2004). Liotti argued that caregiving relationships characterized by simultaneous activation of attachment and fear – as occurs when the caregiver is both a source of comfort and a source of threat or distress – produce a fundamental collapse of the child’s behavioral and representational strategies. In role reversal configurations, while overt fear may be less prominent, a structurally analogous disruption can occur: the child must simultaneously inhibit attachment needs and assume a caregiving function that exceeds developmental capacity. This configuration generates what Liotti described as incoherent internal working models, in which the self is simultaneously experienced as responsible for the other’s wellbeing and as potentially damaging to the relationship by virtue of having needs at all. Such incoherence is precisely what CMT’s construct of pathogenic beliefs seeks to capture at the level of relational meaning.

Among the various forms of attachment-related adversity, role reversal and inverted caregiving are particularly relevant to the present discussion (Hooper, 2007; Hooper et al., 2011). In these configurations, the child gradually assumes responsibilities that exceed normal developmental capacities. Rather than receiving emotional regulation from the caregiver, the child becomes increasingly responsible for monitoring, stabilizing, reassuring, or protecting the caregiver’s emotional state. Although such patterns may emerge in the absence of overt abuse or neglect, they often exert a profound influence on the child’s developing sense of self and relational expectations.

Importantly, the child’s adaptation to these circumstances should not be understood as pathological in itself. Within the original developmental context, heightened sensitivity to the caregiver’s emotional condition may represent an intelligent and adaptive strategy for preserving relational security. Problems arise when these adaptive solutions become generalized beyond the original context and continue to organize adult functioning long after the developmental environment has changed.

Control-Mastery Theory provides a particularly useful framework for understanding how these developmental adaptations become translated into enduring patterns of suffering. According to CMT, children exposed to adverse relational experiences construct pathogenic beliefs that enable them to maintain attachment relationships while making sense of emotionally painful experiences (Weiss, 1993). These beliefs are not irrational distortions but adaptive conclusions derived from the child’s efforts to understand and survive within the interpersonal environment available to them.

In cases involving role reversal and inverted caregiving, pathogenic beliefs frequently center on themes of responsibility, guilt, emotional danger, and relational burden. Patients may develop implicit convictions such as:

- *If I express my needs, I will overwhelm the other person.*
- *If I become dependent, I will damage the relationship.*
- *My feelings are less important than the emotional needs of others.*
- *I am responsible for maintaining the emotional stability of significant people.*
- *If I stop taking care of others, something bad will happen.*

Although these beliefs may appear maladaptive from an adult perspective, they often represent coherent developmental solutions to attachment environments in which the child’s emotional needs were experienced as potentially disruptive or dangerous.

A particularly important implication of this formulation is that the central problem is not simply the persistence of distressing memories, but the persistence of relational meanings attached to those memories. Two individuals may experience similar childhood events yet derive very different conclusions regarding responsibility, dependency, vulnerability, or emotional expression. Consequently, the clinical significance of an attachment-related memory cannot be determined solely by its objective characteristics or emotional intensity. Rather, its significance depends on the role it plays in maintaining the patient’s current pathogenic beliefs.

This observation has important implications for EMDR treatment. In patients presenting with complex attachment histories, clinicians are often confronted with numerous potentially relevant memories. Without a clear conceptual framework, target selection may become driven primarily by symptom intensity, chronological priority, or subjective emotional impact. While such approaches may be clinically useful, they do not necessarily identify the experiences most directly involved in maintaining the patient’s current pathogenic organization.

The present paper proposes that pathogenic beliefs provide the conceptual bridge between attachment trauma and EMDR target selection. Specifically, attachment-related experiences acquire therapeutic relevance to the extent that they continue to sustain the emotional credibility of pathogenic beliefs observable within present functioning. From this perspective, the clinician’s task is not merely to identify traumatic memories, but to understand how particular memories continue to support relational expectations that remain active in the patient’s current life.

This formulation becomes particularly relevant in psychotherapy because pathogenic beliefs do not remain confined to childhood memories. Rather, they emerge repeatedly within contemporary relationships, including the therapeutic relationship itself. As will be discussed in the following section, Control-Mastery Theory conceptualizes testing processes as the primary mechanism through which these beliefs become observable and available for therapeutic disconfirmation. Observation of testing may therefore help clinicians clarify the pathogenic beliefs active in present relational functioning and formulate more focused hypotheses about the attachment-related experiences that may continue to sustain their emotional credibility.

## 3 Control-Mastery Theory and testing processes in attachment- related trauma

Control-Mastery Theory (CMT) proposes that psychopathology is organized around pathogenic beliefs that originate in adverse developmental experiences and continue to shape emotional and interpersonal functioning long after the original circumstances have changed (Weiss, 1993; Weiss and Sampson, 1986). According to this perspective, patients do not enter psychotherapy merely seeking symptom reduction. Rather, they enter treatment with an unconscious plan aimed at overcoming pathogenic beliefs and achieving previously inhibited developmental goals.

A central feature of this model is the concept of testing. In CMT, patients actively and unconsciously test pathogenic beliefs within the therapeutic relationship in order to determine whether feared consequences will continue to occur. Testing is therefore not conceptualized as resistance, alliance rupture, or repetition for its own sake. Instead, it reflects the patient’s effort to obtain new relational information capable of disconfirming pathogenic expectations.

This perspective differs substantially from models that view problematic interpersonal behavior primarily as defensive or maladaptive. While defensive processes undoubtedly play an important role, CMT emphasizes that many apparently difficult interactions within psychotherapy may represent attempts at growth rather than obstacles to treatment. The patient’s behavior can therefore be understood not only in terms of protection from danger, but also in terms of an ongoing search for safety, mastery, and disconfirmation.

This distinction is particularly important in attachment-related trauma. Patients with histories of role reversal and inverted caregiving frequently enter therapy organized around powerful expectations regarding responsibility, emotional burden, dependency, and relational danger. Their pathogenic beliefs often remain implicit and may initially be expressed through subtle interpersonal patterns rather than explicit verbal formulations. As a result, the therapeutic relationship becomes a privileged context in which these beliefs can gradually emerge and become observable.

### 3.1 When testing has not yet begun: defensive caregiving organizations

An important clinical clarification concerns the distinction between testing and defensive relational organization. Not all interpersonal behavior observed in therapy constitutes testing.

Patients with histories of inverted caregiving often enter treatment in a state of heightened monitoring of the therapist’s emotional condition. Sessions may be characterized by excessive politeness, emotional minimization, rapid reassurance of the therapist, reluctance to express disappointment, and persistent concern about taking up too much space. These behaviors may superficially resemble collaboration or alliance, yet their primary function is frequently protective rather than exploratory.

For example, a patient may repeatedly interrupt emotionally significant narratives in order to reassure the therapist that “everything is fine” or may quickly withdraw expressions of anger or sadness out of concern that such emotions could burden the therapist. In these situations, the patient’s behavior is organized primarily around preventing anticipated relational catastrophe rather than testing whether the catastrophe would actually occur.

Clinically, this distinction is crucial. Therapists may mistakenly interpret such behavior as evidence of trust or therapeutic progress when, in fact, the patient remains unable to expose pathogenic beliefs to genuine disconfirmation. Before testing can emerge, the patient must first experience sufficient safety to risk abandoning at least some aspects of the defensive caregiving organization.

### 3.2 Passive-into-active tests

Among the various forms of testing described within CMT, passive-into-active tests are particularly relevant to patients whose developmental histories involve chronic helplessness, role reversal, or emotional subordination.

In passive-into-active testing, the patient reverses the original traumatic relational position by placing the therapist in circumstances analogous to those previously endured by the patient. What defines the test is not the specific behavior itself, but the reversal of the relational configuration.

Patients who grew up feeling responsible for a fragile caregiver may, for example, expose the therapist to criticism, dissatisfaction, emotional pressure, or evaluative scrutiny. Patients who experienced chronic misunderstanding may repeatedly create situations in which the therapist feels confused, inadequate, or uncertain. Others may place the therapist in a position of responsibility for resolving tensions that they themselves were once forced to manage.

The clinical meaning of these interactions is frequently misunderstood. Superficially, they may appear hostile, oppositional, or resistant. However, from a CMT perspective, their deeper function is to determine whether the therapist will reproduce the traumatic response anticipated by the pathogenic belief.

In the context of inverted caregiving, the implicit question often becomes:

*What happens if I stop protecting you?*

Or, more specifically:

*Can you tolerate my disappointment, anger, criticism, or emotional pressure without collapsing, retaliating, withdrawing, or requiring me to take care of you again?*

The therapist’s response to passive-into-active tests is therefore of considerable clinical importance. Defensive explanations, excessive reassurance, subtle retaliatory reactions, or emotional withdrawal may inadvertently confirm the patient’s expectations. Conversely, the therapist’s capacity to tolerate pressure while maintaining reflective functioning may contribute to the gradual disconfirmation of the pathogenic belief.

### 3.3 Transference tests

Transference tests differ from passive-into-active tests in that the patient does not necessarily reverse the original relational position. Instead, the patient recreates conditions under which the therapist is expected to respond similarly to significant attachment figures from the past.

These tests often involve fears of rejection, misunderstanding, criticism, emotional unavailability, or abandonment. For example, a patient may withhold important information while simultaneously anticipating that the therapist will fail to understand them. Another patient may become highly sensitive to minor scheduling changes, interpreting them as evidence of disinterest or rejection.

Unlike passive-into-active tests, the patient’s primary aim is not to place the therapist in the patient’s former position. Rather, the patient is evaluating whether the therapist will confirm the familiar relational pattern predicted by the pathogenic belief.

Transference tests are often subtle and repetitive. Because they may resemble ordinary relational sensitivity, therapists sometimes underestimate their significance. However, careful attention to recurring expectations and emotional reactions often reveals their testing function.

### 3.4 Direct tests

Direct tests generally emerge later in treatment and often indicate increasing psychological safety within the therapeutic relationship.

In direct testing, the patient no longer relies primarily on role reversal or indirect relational maneuvers. Instead, previously inhibited wishes, feelings, and developmental strivings are expressed more openly.

A patient who previously feared burdening others may directly ask for support. A patient organized around fears of rejection may express disappointment without immediately withdrawing. A patient who has long inhibited anger may communicate frustration while remaining emotionally engaged in the relationship.

Importantly, direct tests should not be understood as the absence of anxiety. Patients frequently remain highly apprehensive while engaging in direct testing. What changes is their increasing willingness to tolerate emotional risk in pursuit of disconfirmation.

For this reason, direct tests often provide particularly valuable information regarding readiness for deeper therapeutic work, including trauma processing interventions.

### 3.5 Motivational oscillations and clinical complexity

Patients with attachment-related trauma rarely operate from a single relational organization. Defensive caregiving, attachment needs, self-protective strategies, and testing processes may alternate rapidly within the same session. Consequently, the meaning of a specific behavior cannot be inferred solely from its observable form but must be understood within its relational context and function. Recognizing these shifts is clinically important because they help therapists identify when pathogenic beliefs are becoming increasingly available for observation and disconfirmation. In this way, testing processes provide a bridge between relational understanding and memory-based interventions.

## 4 The therapeutic relationship as a laboratory of pathogenic beliefs

Within the framework proposed here, the therapeutic relationship serves a function that extends beyond the provision of safety, support, or corrective emotional experience. While these elements remain essential, the therapeutic relationship also constitutes the primary context in which pathogenic beliefs become observable through the patient’s ongoing efforts to negotiate attachment, dependency, autonomy, responsibility, and emotional risk.

From a Control-Mastery perspective, pathogenic beliefs are not merely cognitive propositions stored within memory. Rather, they are living relational expectations that continuously organize interpersonal behavior. Consequently, their influence becomes most apparent not when patients describe past experiences, but when they encounter situations in the present that activate the same fears, conflicts, and expectations that originally contributed to their development.

This observation is particularly relevant in attachment-related trauma. Patients frequently possess considerable insight into their developmental histories and may be capable of describing childhood experiences with remarkable clarity. However, intellectual awareness alone does not necessarily provide access to the pathogenic meanings that continue to organize current functioning. In many cases, the most clinically significant beliefs become visible only when they are enacted, tested, feared, or defended against within the therapeutic relationship itself.

For this reason, the therapeutic relationship can be understood as a laboratory in which pathogenic beliefs become progressively observable. Through testing processes, patients create situations that reveal not only what they fear, but also what they expect to occur when they pursue previously inhibited developmental goals. The therapist’s task is therefore not limited to understanding the patient’s history; it also involves recognizing how the patient’s history continues to organize present relational experience.

### 4.1 From emotional activation to clinical observability

One of the central arguments of this paper is that emotional activation should not be confused with clinical observability. Patients may experience intense affect when discussing attachment-related memories, yet such activation does not necessarily reveal the pathogenic beliefs through which those experiences continue to influence present functioning.

Clinical observability emerges when these beliefs become visible within current relational behavior. Through testing processes, defensive organizations, and recurring interpersonal expectations, pathogenic beliefs become accessible not merely as retrospective explanations but as active organizing principles of present experience.

This distinction has important implications for EMDR case conceptualization. Emotional intensity alone does not necessarily indicate which memories are most relevant to treatment. Rather, clinicians must understand how specific attachment experiences continue to support pathogenic beliefs that remain active within the patient’s relational world.

From this perspective, the therapeutic relationship serves not only as a source of safety but also as a source of information. Through testing processes, patients reveal the expectations through which they interpret emotional experience and interpersonal relationships. Observation of these processes may help the therapist clarify which pathogenic beliefs remain operative and formulate hypotheses about the attachment-related experiences that continue to sustain their emotional credibility.

The therapeutic relationship may therefore function as a bridge between present relational functioning and developmental attachment experiences, helping clinicians refine hypotheses about potentially relevant memories for subsequent EMDR assessment and processing.

### 4.2 Relational disconfirmation and the transformation of meaning

Control-Mastery Theory proposes that therapeutic change occurs through the gradual disconfirmation of pathogenic beliefs. Importantly, disconfirmation does not occur through persuasion, interpretation, or reassurance alone. Rather, it emerges through repeated interpersonal experiences in which anticipated relational catastrophes fail to occur.

For patients organized around inverted caregiving, this process often involves discovering that emotional needs do not inevitably damage the relationship, that disappointment can be tolerated without retaliation, and that dependency does not necessarily result in collapse or abandonment.

The significance of these experiences should not be underestimated. Relational disconfirmation alters the patient’s relationship to the pathogenic belief itself. Beliefs that previously appeared self-evident begin to acquire a degree of uncertainty. Alternative interpretations become conceivable. Previously feared behaviors become increasingly imaginable.

Importantly, however, relational disconfirmation does not automatically eliminate the emotional force of the pathogenic belief. Patients frequently continue to experience powerful emotional reactions even after recognizing that the therapist has repeatedly contradicted their expectations.

This observation is particularly important for understanding the potential role of EMDR within the integrative model proposed here.

### 4.3 Common clinical errors

Several clinical errors may interfere with the observation and disconfirmation of pathogenic beliefs in attachment-related trauma.

A first error consists of mistaking defensive caregiving for therapeutic alliance. Patients who appear highly collaborative, insightful, and considerate may still be organized around profound fears concerning emotional burden and relational danger. In such cases, apparent cooperation may conceal the absence of genuine testing.

A second error involves misunderstanding passive-into-active tests as evidence of hostility, resistance, or treatment rupture. When therapists respond defensively to criticism, evaluative pressure, or dissatisfaction, they may inadvertently confirm precisely the expectations the patient is attempting to test.

A complementary error is to assume that criticism, dissatisfaction, or relational tension necessarily constitutes a test. Some interactions may instead reflect legitimate complaints, therapist errors, or boundary problems and require direct acknowledgement and repair. Conversely, when an interaction does constitute a test, a therapist response that confirms the feared pathogenic expectation may result in a failure of the test and contribute to an alliance rupture. The distinction cannot be established from observable behavior alone, but requires attention to the relational context, recurrent function, the patient’s developmental formulation, and the therapist’s own contribution to the interaction. A CMT-informed formulation should therefore remain tentative and should not be used to minimize legitimate dissatisfaction or to exempt the therapist from clinical responsibility.

A third error involves excessive reassurance. Although reassurance is often well-intentioned, premature reassurance may interrupt testing processes by reducing opportunities for patients to discover, through experience, that feared relational outcomes need not occur.

Finally, clinicians may prematurely select EMDR targets on the basis of emotional intensity alone. While highly distressing memories are often important, symptom intensity does not necessarily correspond to structural relevance. Some of the most clinically significant attachment memories are not those associated with the greatest emotional activation, but those that continue to provide emotional credibility to pathogenic beliefs observable in present relational functioning.

For this reason, careful observation of testing processes may contribute not only to relational understanding but also to more precise and theoretically informed decisions regarding EMDR intervention.

The following section develops this argument further by examining how relational disconfirmation may increase readiness for memory processing and how pathogenic beliefs may guide the selection of EMDR targets in attachment-related trauma.

## 5 From relational disconfirmation to EMDR processing: a control-mastery perspective

The integration proposed in this paper rests on the assumption that relational disconfirmation and memory processing represent distinct but complementary mechanisms of therapeutic change. While Control-Mastery Theory and EMDR originate from different theoretical traditions, both models are concerned with the persistence of maladaptive expectations derived from adverse experiences and with the conditions necessary for their transformation.

Control-Mastery Theory focuses primarily on the relational dimension of change. Therapeutic progress occurs as patients test pathogenic beliefs and gradually discover that anticipated relational catastrophes do not inevitably occur. Through repeated experiences of disconfirmation, beliefs that previously appeared unquestionable begin to lose their certainty and organizing power.

Eye Movement Desensitization and Reprocessing (EMDR), by contrast, focuses primarily on the processing of maladaptively stored experiences. According to the Adaptive Information Processing (AIP) model, psychopathology is maintained when memories remain insufficiently integrated within adaptive memory networks and continue to influence present perception, affect regulation, and behavior (Shapiro, 2001, 2018).

The present model proposes that these two perspectives may be understood as addressing different aspects of the same clinical process.

### 5.1 Why relational disconfirmation alone may not be sufficient

Clinical experience suggests that patients frequently demonstrate meaningful relational change before corresponding emotional changes become fully consolidated.

For example, a patient may recognize that the therapist has repeatedly tolerated disappointment, dependency, criticism, or emotional need without withdrawing or retaliating. At an explicit level, the patient may acknowledge that the feared outcome has not occurred. Nevertheless, situations involving intimacy, vulnerability, or emotional dependence may continue to evoke intense anxiety, guilt, shame, or anticipatory fear.

This apparent discrepancy raises an important clinical question: why do pathogenic beliefs often retain emotional force even after substantial relational disconfirmation has occurred?

From the perspective proposed here, one possible explanation is that relational disconfirmation and memory integration operate at different levels of the therapeutic process. Relational experiences may challenge the validity of the pathogenic belief, but the attachment-related memories that originally conferred emotional credibility upon that belief may continue to remain insufficiently integrated.

Consequently, patients may intellectually recognize that a belief is inaccurate while continuing to experience it as emotionally true.

A patient may understand that expressing needs does not inevitably overwhelm others and yet continue to experience intense anxiety whenever dependency becomes emotionally salient. Another may recognize that anger can be tolerated within relationships while still experiencing profound guilt when attempting to assert personal boundaries.

Such observations suggest that relational disconfirmation, although essential, may not always be sufficient to modify the emotional foundations of pathogenic beliefs.

### 5.2 Why EMDR may become particularly relevant at this stage

Within the framework proposed here, EMDR becomes especially relevant when pathogenic beliefs have become sufficiently explicit and observable through testing processes, yet continue to derive emotional credibility from unresolved attachment-related experiences.

At this point, the therapist possesses information that was not necessarily available earlier in treatment.

Through observation of testing processes, the clinician has gained increasing clarity regarding:

- the central pathogenic belief,
- the developmental conflicts organized around that belief,
- the relational situations that activate it,
- and the attachment-related experiences most likely to have contributed to its formation.

The role of EMDR is therefore not to discover the pathogenic belief but to process the experiences that continue to sustain its emotional plausibility.

This distinction is important because it positions EMDR within a broader developmental sequence rather than as an isolated intervention. Memory processing becomes informed by relational understanding, while relational understanding becomes consolidated through memory processing.

### 5.3 Relational readiness for memory processing

A central concept emerging from the present integration is that of relational readiness. This construct is not intended to replace established considerations of readiness, stabilization, affect tolerance, or resource availability within EMDR treatment. Preparation for memory processing has long included attention to the patient’s capacity to maintain sufficient emotional regulation and access adaptive resources, and Resource Development and Installation (RDI) was specifically developed to strengthen such capacities in complex trauma presentations (Korn and Leeds, 2002; Hase, 2021). More broadly, phase-oriented approaches to complex trauma have emphasized safety, stabilization, affect regulation, and the development of sufficient internal and interpersonal resources before or alongside trauma-focused work.

Relational readiness is proposed here as a complementary, rather than alternative, dimension of case conceptualization. It refers specifically to the extent to which a pathogenic relational expectation has become sufficiently observable within the therapeutic relationship for the patient and therapist to recognize how it continues to organize present experience. In attachment-related trauma, a patient may possess adequate affect-regulation capacities and nevertheless remain strongly organized around defensive relational strategies that prevent central pathogenic expectations from becoming accessible to observation and disconfirmation. Conversely, the emergence of relational risk-taking may occur while substantial anxiety remains present.

From this perspective, relational readiness does not indicate that a patient is globally “ready” or “not ready” for EMDR, nor is it proposed as a mandatory preliminary phase before memory processing. Rather, it describes a clinically observable dimension that may contribute to decisions about timing and target formulation when attachment-related pathogenic beliefs are central to the case. Its proposed added value lies in directing attention not only to whether the patient can tolerate memory processing, but also to whether the relational meaning linking current difficulties to developmental experiences has become sufficiently explicit to inform the formulation of candidate targets.

Several clinical indicators may suggest increasing relational readiness:

- reduction in defensive caregiving toward the therapist;
- increased tolerance for emotional proximity;
- emergence of passive-into-active and direct tests;
- greater capacity to express disappointment, anger, dependency, or need;
- reduced fear of destabilizing the therapist;
- increasing explicitness of the pathogenic belief;
- greater ability to reflect on relational experiences without immediate defensive retreat.

Importantly, these indicators should not be interpreted as prerequisites that must be achieved before EMDR processing can occur. The empirical literature does not support a universal requirement for prolonged stabilization before trauma-focused treatment in all complex trauma presentations (de Jongh et al., 2016; van Vliet et al., 2021). Accordingly, relational readiness is advanced here as a theoretical and clinically derived construct whose incremental value, reliability, and relationship to established readiness criteria require empirical investigation.

### 5.4 A pathogenic belief-guided model of EMDR target selection

One of the most clinically relevant implications of this integration concerns the selection of EMDR targets.

In complex attachment trauma, clinicians are often confronted with numerous potentially relevant memories. Without a guiding framework, target selection may become based primarily on emotional intensity, chronological priority, or subjective salience.

The present model proposes an alternative approach.

Rather than asking:

*“Which memory is most traumatic?”*

the clinician asks:

*“Which memories continue to support the emotional credibility of the pathogenic belief currently observable within the patient’s relational functioning?”*

This shift has important implications.

Attachment-related memories are not selected simply because they are painful. They are selected because they continue to function as evidence supporting a pathogenic expectation that remains active in the present.

In this sense, the therapeutic relationship becomes an additional source of information for formulating hypotheses about potentially relevant memories. Testing processes may help clarify the pathogenic beliefs currently organizing the patient’s difficulties; those beliefs, in turn, provide a relationally informed criterion for exploring which developmental experiences continue to sustain their emotional credibility. The relationship therefore does not determine which memories re quire processing, but may contribute clinically meaningful information to be integrated with AIP-informed history-taking and standard EMDR target assessment.

### 5.5 An integrative model of therapeutic change

The model proposed here conceptualizes therapeutic change as a dynamic interaction between relational disconfirmation and memory reprocessing.

Testing processes render pathogenic beliefs increasingly visible.

Relational disconfirmation weakens the certainty of those beliefs.

EMDR processes the attachment-related experiences that continue to support their emotional credibility.

As these memories become integrated within adaptive memory networks, the pathogenic belief loses not only cognitive plausibility but also emotional conviction.

From this perspective, EMDR and Control-Mastery Theory should not be understood as competing explanations of therapeutic change. Rather, they may be viewed as complementary perspectives addressing different dimensions of the same developmental process.

The therapeutic relationship facilitates the emergence and disconfirmation of pathogenic beliefs; EMDR facilitates the integration of the attachment-related experiences that continue to sustain them.

Together, these processes may offer a particularly useful framework for understanding and treating attachment-related trauma, especially in patients whose developmental histories have been characterized by role reversal, inverted caregiving, and chronic responsibility for the emotional wellbeing of others.

## 6 A Control-Mastery-Informed model of EMDR target selection

The theoretical integration proposed in this paper has practical implications for one of the most challenging aspects of EMDR treatment in attachment-related trauma: target selection.

In straightforward trauma presentations, target identification is often relatively uncomplicated. A specific traumatic event can be identified, linked to current symptoms, and processed according to established EMDR protocols. In attachment-related trauma, however, clinicians frequently encounter a more complex situation. Patients may report numerous painful memories distributed across many years of development, with no single event appearing obviously central to current difficulties.

Under these circumstances, target selection becomes less a matter of identifying the most distressing memory and more a matter of identifying the memories that continue to organize present functioning.

The model proposed here suggests that Control-Mastery Theory may offer a clinically useful pathway for addressing this challenge.

### 6.1 A clinical sequence for EMDR target selection

The integration proposed here unfolds as a clinically guided sequence, but testing should not be understood as a procedure that directly identifies specific autobiographical memories. The clinician initially develops a tentative formulation of the patient’s central symptoms, interpersonal difficulties, developmental history, and possible pathogenic beliefs. Observation of testing processes within the therapeutic relationship then provides an additional source of information through which this formulation can be refined. Recurrent relational patterns–such as the patient placing the therapist in a particular interpersonal position, anticipating a specific response, or progressively risking previously inhibited needs–may help clarify which pathogenic belief is currently organizing the patient’s experience.

The contribution of testing therefore lies primarily in **constraining and refining the clinical hypothesis,** rather than in generating an EMDR target independently of standard case conceptualization. Once a pathogenic belief has become sufficiently explicit, the clinician returns to the patient’s developmental and autobiographical history and asks which experiences contributed to establishing, reinforcing, or maintaining the emotional credibility of that belief. This process may generate a set of candidate memories sharing a common relational meaning even when they differ substantially in objective severity or subjective distress.

Standard AIP-informed history-taking and EMDR target assessment remain necessary for evaluating these candidate memories and determining their appropriateness for processing. CMT-informed observation is proposed as complementary to, rather than a replacement for, these procedures. Its specific contribution is to provide relationally derived information about the meaning currently linking past experiences to present difficulties. Accordingly, target selection emerges from the convergence of developmental history, current symptoms, AIP-informed assessment, and the pathogenic belief as it becomes observable within the therapeutic relationship.

Processing can then proceed while maintaining awareness of the relationship between the selected memory, the pathogenic belief, and its current relational manifestations. In this way, memory work remains anchored within the broader case formulation without assuming that testing alone can establish which memory constitutes the clinically appropriate EMDR target.

The proposed clinical sequence and the complementary contributions of relational disconfirmation and memory processing are summarized in Figure 1 .

**FIGURE 1:**
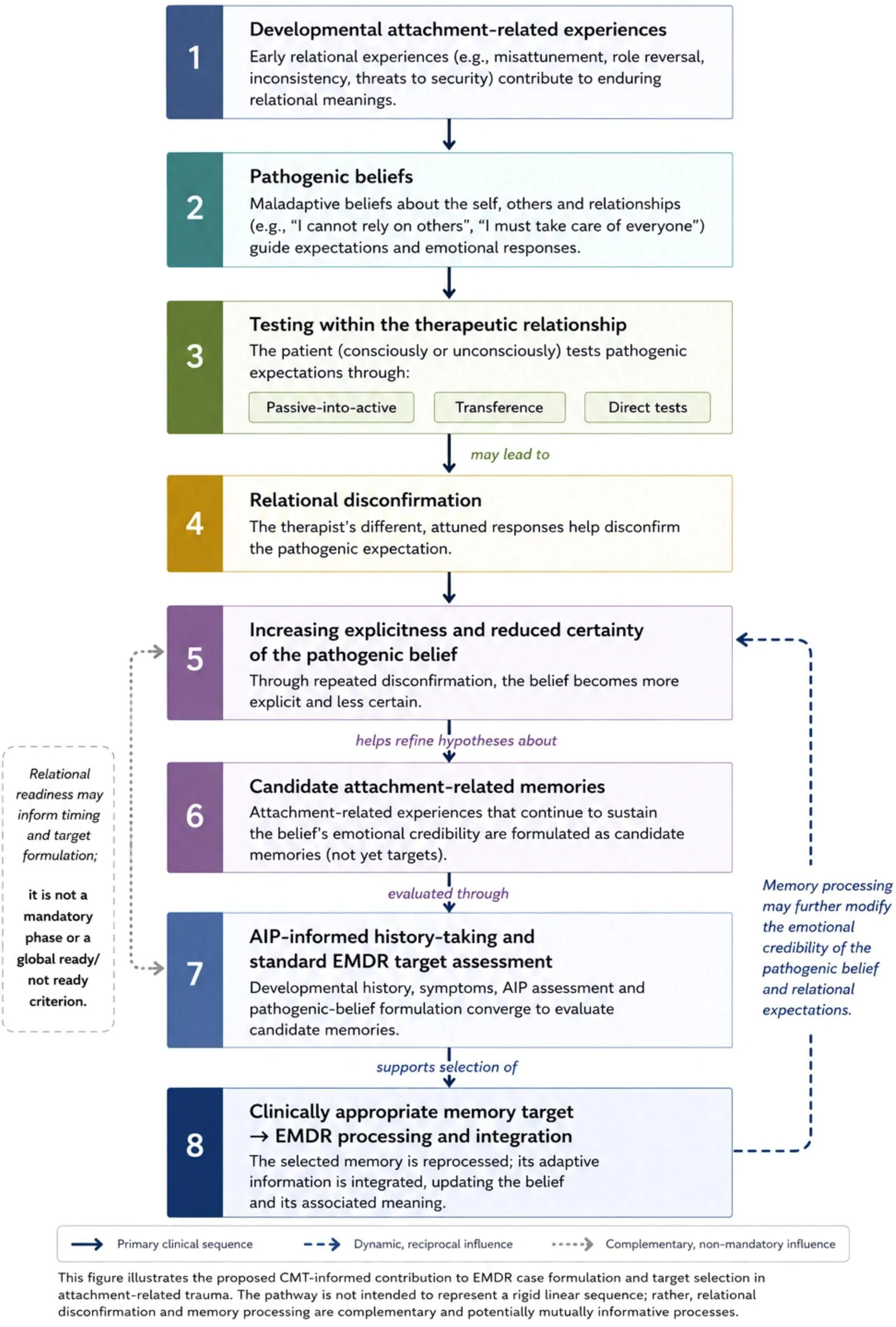
Proposed CMT-informed pathway from relational testing to EMDR target formulation and memory processing.

### 6.2 Clinical advantages of the model

Several potential advantages emerge from this approach.

First, it reduces the risk of selecting targets solely on the basis of symptom intensity. Although emotional activation remains important, intensity alone may not accurately indicate structural relevance.

Second, it promotes greater integration between psychotherapy process and EMDR intervention. Rather than functioning as separate components of treatment, relational work and memory processing become mutually informative.

Third, it may be particularly useful in complex attachment trauma, where developmental experiences are often numerous, cumulative, and difficult to prioritize.

Finally, the model introduces relational readiness as an additional consideration that may inform the timing and formulation of EMDR interventions in attachment-related presentations. This dimension complements, rather than replaces, established considerations of affect regulation, stabilization, resource availability, and standard EMDR readiness assessment by considering the extent to which relevant pathogenic beliefs have become observable within the therapeutic relationship.

In this sense, target selection becomes less a technical decision and more the outcome of an evolving understanding of the patient’s developmental history, pathogenic beliefs, and testing processes.

The following clinical illustration demonstrates how these principles may operate in practice and how the progression from testing to memory processing can inform therapeutic decision-making in a patient whose developmental history was characterized by role reversal and inverted caregiving.

## 7 Clinical illustration

### 7.1 Clinical presentation and initial formulation

The following clinical illustration is intended to demonstrate how the integration of Control-Mastery Theory and EMDR may inform case conceptualization and target selection in attachment-related trauma. The case has been modified to protect confidentiality while preserving the clinical processes relevant to the present discussion.

The patient was a woman in her late thirties who sought psychotherapy due to chronic relational anxiety, pervasive guilt, difficulties expressing emotional needs, and recurrent feelings of responsibility for the wellbeing of others. Although professionally successful and generally perceived by others as competent and emotionally stable, she reported persistent exhaustion in intimate relationships and a longstanding tendency to prioritize the needs of others over her own.

Her developmental history was characterized by a chronically fragile and emotionally overwhelmed mother. The patient described her childhood not in terms of overt abuse or neglect, but rather as a continuous effort to monitor and manage her mother’s emotional state. She recalled becoming highly sensitive to subtle changes in mood, learning to suppress her own distress when her mother appeared upset, and experiencing intense guilt whenever she perceived herself as creating additional difficulties.

At intake, the patient’s difficulties were initially formulated as reflecting an attachment-related pathogenic belief organized around responsibility and emotional burden. Although the belief was not yet fully explicit, the preliminary formulation suggested the presence of expectations such as:

*“My needs place excessive demands on others.”*

“If I become emotionally dependent, I will overwhelm the people I love.”

*“I am responsible for maintaining relational stability.”*

These hypotheses remained tentative and required further observation within the therapeutic relationship.

### 7.2 Defensive caregiving organization and the pre-testing phase

During the initial phase of treatment, the patient did not appear to engage in testing processes in the strict Control-Mastery sense. Instead, the therapeutic relationship was characterized by a pervasive caregiving organization.

She consistently monitored the therapist’s reactions, frequently interrupted emotionally significant narratives to minimize their impact, and often expressed concern about “taking up too much space” during sessions. When discussing painful experiences, she regularly shifted attention away from her own emotions and toward the presumed emotional experience of others.

For example, after describing a particularly distressing interpersonal conflict, she quickly added:

*“But I can also understand why she reacted that way. She was under a lot of pressure.”*

Similarly, after discussing experiences of loneliness within an intimate relationship, she immediately redirected attention toward her partner’s difficulties and vulnerabilities.

At first glance, these interactions could easily have been interpreted as evidence of insight, empathy, or mature reflective functioning. However, repeated observation suggested that their primary function was defensive. The patient’s emotional attention remained focused not on her own experience but on preventing potential distress in others.

A similar pattern emerged in the therapeutic relationship. Expressions of frustration, disappointment, dependency, or emotional need were often followed by attempts to reassure the therapist or minimize the significance of what had just been disclosed.

From a Control-Mastery perspective, these interactions were understood not as testing but as manifestations of a defensive caregiving organization developed in response to chronic role reversal during childhood.

Importantly, the absence of overt conflict should not have been mistaken for therapeutic safety. The patient remained unable to risk exposing the pathogenic belief to genuine relational disconfirmation because she continued to organize herself around protecting the therapist from emotional burden.

### 7.3 Emergence of passive-into-active tests

As treatment progressed and the therapeutic relationship became increasingly stable, subtle shifts began to emerge.

The patient gradually reduced her constant monitoring of the therapist and became more willing to express dissatisfaction, irritation, and disappointment. These changes initially appeared modest but represented an important transformation in relational organization.

A recurring pattern emerged following sessions in which emotionally significant material had been discussed. The patient would return the following week appearing visibly tense and critical of the previous session.

On several occasions she stated that the therapist had “missed something important” or had failed to understand the emotional significance of what she had attempted to communicate.

At times she suggested that the therapist’s interventions had been too intellectual, insufficiently empathic, or emotionally detached.

Although these interactions could easily have been interpreted as ordinary dissatisfaction or alliance fluctuations, their function became increasingly apparent over time.

The patient appeared to place the therapist in a position characterized by uncertainty, inadequacy, and evaluative pressure. During such exchanges, the therapist occasionally experienced transient feelings of self-doubt and subtle pressure to repair the interaction quickly.

Importantly, the patient was no longer primarily concerned with protecting the therapist.

Instead, she appeared to be exploring what would happen if she stopped doing so.

These interactions were conceptualized as passive-into-active tests.

The original developmental configuration had involved the patient occupying the position of the emotionally responsible child confronted with a vulnerable caregiver. Through passive-into-active testing, this configuration was partially reversed.

The therapist was now exposed to feelings of inadequacy, pressure, and concern regarding relational failure that resembled aspects of the patient’s earlier developmental experience.

The central relational question appeared to shift from:

*“Will my needs hurt you?”*

to:

*“What happens if I stop protecting you from my disappointment?”*

The therapist’s responses during this phase proved clinically significant.

Attempts to reassure the patient prematurely often seemed to restore the original caregiving configuration. When reassurance occurred too quickly, the patient frequently returned to monitoring the therapist and minimizing her own emotional experience.

By contrast, therapeutic progress was facilitated when the therapist was able to tolerate criticism, disappointment, and evaluative pressure without becoming defensive, withdrawing emotionally, or requiring reassurance from the patient.

Repeated experiences of this kind gradually weakened the patient’s expectation that authentic emotional impact would inevitably destabilize the relationship.

### 7.4 Emergence of direct tests and increasing relational risk

A further shift became evident when the patient began expressing emotional needs more directly.

Rather than relying primarily on criticism or evaluative pressure, she increasingly disclosed fears concerning dependency, rejection, and emotional burden.

In one session, after a prolonged silence, she remarked:

*“Sometimes I think that if people really knew how much I need from them, they would eventually become exhausted.”*

Unlike earlier phases of treatment, this statement was not immediately followed by minimization or attempts to protect the therapist from its emotional implications.

Instead, the patient remained engaged while visibly anxious, apparently observing whether the therapist would confirm or disconfirm the feared expectation.

This moment represented an important transition toward direct testing.

The pathogenic belief became increasingly explicit:

“If I become fully real in a relationship, I will eventually overwhelm the other person.”

Although significant anxiety remained present, the patient was becoming increasingly capable of tolerating emotional exposure without immediately retreating into defensive caregiving.

The therapeutic relationship had begun to provide sufficient evidence that alternative relational outcomes were possible.

### 7.5 EMDR target selection and processing

It was at this stage that EMDR became clinically relevant within the framework proposed in this paper.

Importantly, target selection was not guided primarily by symptom intensity or by the chronological identification of the most painful childhood memory.

Instead, the selection process was informed by the pathogenic belief that had gradually emerged through testing.

The clinical question shifted from:

*“Which memory is most upsetting?”*

to:

*“Which experiences continue to make this belief feel emotionally true?”*

Several potentially relevant childhood memories were identified. However, particular significance was attributed to a series of experiences in which the patient’s expressions of distress coincided with visible emotional collapse, withdrawal, or dysregulation in her mother.

Although none of these experiences would necessarily have qualified as conventionally traumatic events, they appeared highly relevant to the development of the belief that emotional needs place excessive demands on significant others.

EMDR processing focused on these attachment-related experiences and on the meanings associated with them.

The patient’s initial subjective distress associated with memories involving inadequacy and shame was consistently high, with SUD ratings typically in the 7–8 range. Over successive sets of bilateral stimulation, distress progressively decreased, generally reaching approximately 3–4 as processing advanced. This reduction was accompanied by a change in the subjective meaning of the memories: feelings of shame and personal inadequacy became less dominant, while more adaptive perspectives concerning responsibility, self-worth, and relational safety gradually emerged.

These values are reported as approximate clinical ratings derived from the therapeutic process rather than as systematically collected research data. The vignette is therefore intended to illustrate the course of EMDR processing and its relationship to the proposed case formulation, rather than to provide quantitative evidence of treatment efficacy.

Throughout processing, the therapist maintained awareness of the relationship between the targeted memories and the pathogenic belief that had emerged within therapy.

This allowed memory processing to remain anchored within the broader therapeutic formulation rather than becoming disconnected from the patient’s ongoing relational experience.

### 7.6 Post-processing changes

Following EMDR processing, several clinically meaningful changes became apparent.

The patient reported a reduction in chronic guilt associated with expressing emotional needs and described greater freedom in seeking support from significant others.

Interpersonally, she appeared less preoccupied with monitoring the emotional condition of others and more capable of recognizing her own needs without immediate self-criticism.

Changes also became evident within the therapeutic relationship.

Passive-into-active tests became less frequent and less emotionally charged. Direct expressions of disappointment, dependency, and emotional need increasingly occurred without subsequent retreat into defensive caregiving.

Importantly, the patient continued to experience vulnerability and relational anxiety. However, these experiences appeared less strongly organized around expectations of inevitable emotional burden and relational damage.

### 7.7 Clinical implications

This clinical illustration highlights the potential value of integrating Control-Mastery Theory and EMDR in the treatment of attachment-related trauma.

More specifically, it demonstrates how testing processes may provide clinically useful information regarding the pathogenic beliefs organizing present suffering and how such information may subsequently guide EMDR target selection.

Within this framework, the therapeutic relationship does not merely create the conditions for memory processing. It may also provide clinically meaningful information that, when integrated with AIP-informed history-taking and standard EMDR assessment, helps refine hypotheses about which attachment-related memories remain relevant to the patient’s current pathogenic organization.

The integration proposed here therefore suggests a reciprocal relationship between relational disconfirmation and memory processing, in which each process may inform and support the other within the broader course of treatment.

## 8 Discussion

The present paper has proposed a clinical-theoretical integration between Control-Mastery Theory and EMDR for the treatment of attachment-related trauma. The central proposition is that testing processes within the therapeutic relationship may provide clinically meaningful information regarding the pathogenic beliefs that organize present suffering. This information may, in turn, refine hypotheses about potentially relevant attachment-related memories, whose suitability as EMDR targets remains to be established through AIP-informed history-taking and standard EMDR target assessment. The primary contribution of this model lies in its attempt to bridge relational formulation and memory processing. While EMDR offers a powerful framework for understanding how inadequately processed experiences continue to influence present functioning, Control-Mastery Theory provides a clinically observable pathway through which therapists may identify the beliefs that connect developmental experiences to current symptoms. In this sense, pathogenic beliefs function as a conceptual bridge linking attachment trauma, present relational functioning, and memory-based intervention.

The proposed integration nevertheless requires acknowledgment of an important metatheoretical difference between CMT and the AIP model. CMT is explicitly motivational and intentional in its account of psychological functioning: patients are understood as pursuing adaptive goals and, through an unconscious plan, actively testing pathogenic beliefs in order to seek experiences that may disconfirm them (Weiss, 1993; Weiss and Sampson, 1986). The AIP model, by contrast, is primarily an information-processing account in which current symptoms are understood in relation to maladaptively stored experiences and their insufficient integration within adaptive memory networks (Shapiro, 2001, 2018). It does not require the assumption that relational behavior is organized by an unconscious plan or directed toward the active testing of pathogenic beliefs.

The present proposal therefore does not assume theoretical equivalence between the two models or attempt to reconcile their different metatheoretical commitments. Rather, it treats them as potentially complementary at the level of clinical formulation. CMT is used to generate hypotheses about the motivational and relational meaning of current interpersonal processes, whereas AIP provides the framework through which relevant memory networks are assessed and processed. The integration proposed here is consequently pragmatic and hypothesis-generating rather than a claim that CMT and AIP constitute a unified explanatory theory. Whether CMT-derived constructs such as testing and pathogenic beliefs provide incremental clinical or predictive value beyond established AIP case conceptualization remains an empirical question.

A related point of comparison concerns the Theory of Structural Dissociation. Some of the clinical phenomena described in the present model–including shifts between defensive caregiving, attachment needs, self-protective strategies, and different forms of relational testing–may phenomenologically resemble changes in action systems or dissociative parts described within structural dissociation frameworks (van der Hart et al., 2006). The conceptual frameworks, however, should not be treated as interchangeable. Structural dissociation theory explains such shifts primarily in terms of divisions within personality associated with trauma-related action systems, whereas CMT interprets testing in functional and motivational terms, asking what pathogenic belief is being examined and what feared consequence the patient may be attempting to disconfirm. The same clinical behavior may therefore receive different explanatory interpretations within the two models. The present proposal does not attempt to adjudicate between these accounts; rather, it recognizes structural dissociation as an important alternative framework, particularly in patients presenting with significant dissociative symptoms, for whom assessment of dissociation and appropriate treatment planning remain essential.

The model further suggests that the therapeutic relationship contributes more than safety and emotional support. Through testing processes, patients reveal expectations concerning dependency, responsibility, emotional burden, rejection, and interpersonal safety. These interactions may provide clinically useful information about the relational meanings currently associated with pathogenic beliefs and may thereby contribute to the formulation of hypotheses about potentially relevant attachment-related experiences. Such hypotheses require further evaluation through standard AIP-informed history-taking and EMDR target assessment before memories are selected for processing. A related implication concerns EMDR target selection. In complex attachment trauma, clinicians are often confronted with multiple potentially significant developmental experiences. The present model proposes that target selection may be guided not simply by emotional intensity but by the extent to which specific memories continue to support the pathogenic beliefs observable within current relational functioning.

The concept of relational readiness was introduced to describe the patient’s increasing capacity to engage with pathogenic beliefs as present relational realities rather than solely as historical narratives. Although this concept remains theoretical, it may provide an additional dimension for considering the timing and formulation of trauma processing even when significant anxiety remains present. Several limitations should be acknowledged. The model is conceptual and clinically derived rather than empirically validated. The relationships proposed between testing processes, pathogenic beliefs, and EMDR target selection require systematic investigation. Furthermore, the clinical illustration presented here is intended to demonstrate theoretical principles rather than establish treatment efficacy. Future research may explore whether specific forms of testing predict treatment outcome, whether relational readiness can be operationalized reliably, and whether pathogenic belief-guided target selection contributes to improved clinical results.

Despite these limitations, the integration proposed here offers a potentially useful framework for clinicians working with attachment-related trauma. By conceptualizing testing processes as a source of information regarding pathogenic beliefs and structurally relevant memories, it seeks to strengthen the connection between relational understanding and memory processing within EMDR treatment.

## 9 Conclusion

Attachment-related trauma presents unique challenges for both psychotherapy and EMDR treatment. Unlike single-incident trauma, developmental attachment experiences often involve cumulative relational processes that become organized around enduring pathogenic beliefs concerning dependency, responsibility, emotional expression, and interpersonal safety. As a result, identifying clinically meaningful targets for memory processing may be considerably more complex than simply locating the most emotionally distressing experiences.

The present paper has proposed a clinical-theoretical integration between Control-Mastery Theory and EMDR that seeks to address this challenge. More specifically, it has argued that testing processes within the therapeutic relationship may provide a clinically useful pathway for identifying the pathogenic beliefs that organize a patient’s current suffering and for refining hypotheses about the attachment-related experiences that may continue to sustain the emotional credibility of those beliefs.

Within this framework, pathogenic beliefs occupy a central position. They connect developmental attachment experiences with present relational functioning and provide a conceptual bridge between psychotherapy process and memory processing. Through the observation of testing processes, therapists may gain clinically useful information about the relational meanings that remain active in the patient’s current life. These meanings, in turn, may help refine hypotheses about potentially relevant attachment-related experiences, to be evaluated alongside AIP-informed history-taking and standard EMDR target assessment when selecting memories for processing. The model further suggests that readiness for memory processing may involve more than affect regulation and symptom stability alone. In attachment-related trauma, increasing capacity to expose, test, and tolerate pathogenic beliefs within the therapeutic relationship may represent an important aspect of readiness for deeper trauma processing. In this sense, relational disconfirmation and memory reprocessing can be understood as complementary components of therapeutic change rather than competing explanations of it.

From this perspective, EMDR is not viewed simply as a technique for reducing symptoms associated with traumatic memories. Rather, it becomes part of a broader therapeutic process in which relational observation, testing, disconfirmation, and memory integration interact to promote psychological change.

Ultimately, the central proposition of this paper is that the therapeutic relationship may contribute not only to the conditions supporting EMDR processing, but also to the clinical formulation of the pathogenic beliefs linking present relational difficulties to attachment-related experiences. When integrated with established EMDR case conceptualization and target assessment, this relational information may contribute to a more informed selection of memories for processing. By integrating these relational and memory-based dimensions of treatment, clinicians may be better equipped to conceptualize, target, and treat the enduring effects of attachment-related trauma.

## Data Availability

The original contributions presented in this study are included in this article/supplementary material, further inquiries can be directed to the corresponding author.
